# Supplementary material for: Human BioMolecular Atlas Program (HuBMAP): 3D Human Reference Atlas Construction and Usage
Source: bioRxiv. 2024 Aug 14:2024.03.27.587041. Preprint. [Version 3] doi: 10.1101/2024.03.27.587041 (PMC11142047; doi:10.1101/2024.03.27.587041)
Supplement: Supplement 1 [file media-1.zip › 13 v3.12.2024.pdf]

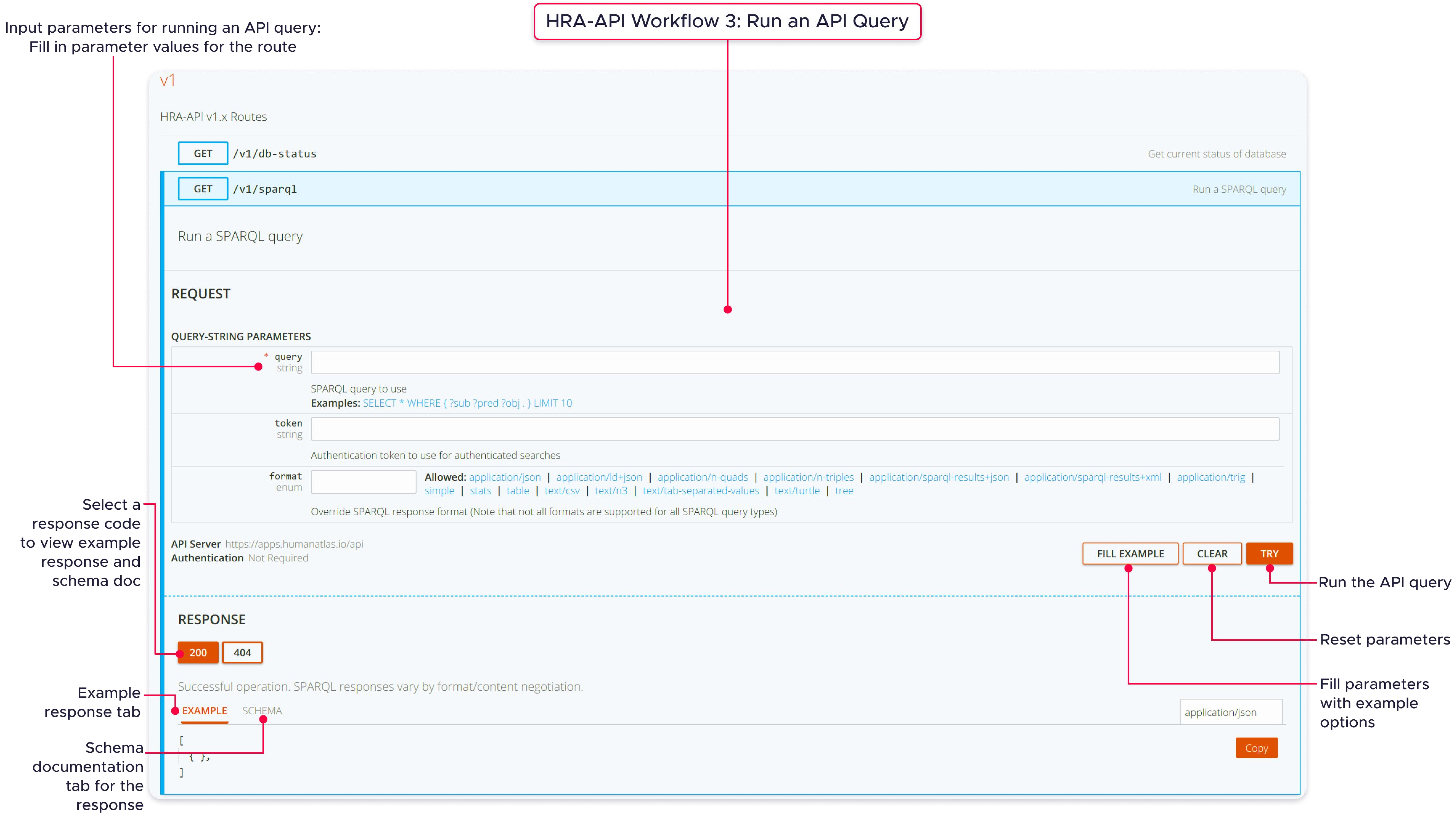

Supplemental Figure 13: Human Reference Atlas Application Programming Interface: Run an API Query
