# Supplementary figures and images for: Human BioMolecular Atlas Program (HuBMAP): 3D Human Reference Atlas Construction and Usage
Source: bioRxiv. 2024 Aug 14:2024.03.27.587041. Preprint. [Version 3] doi: 10.1101/2024.03.27.587041 (PMC11142047; doi:10.1101/2024.03.27.587041)

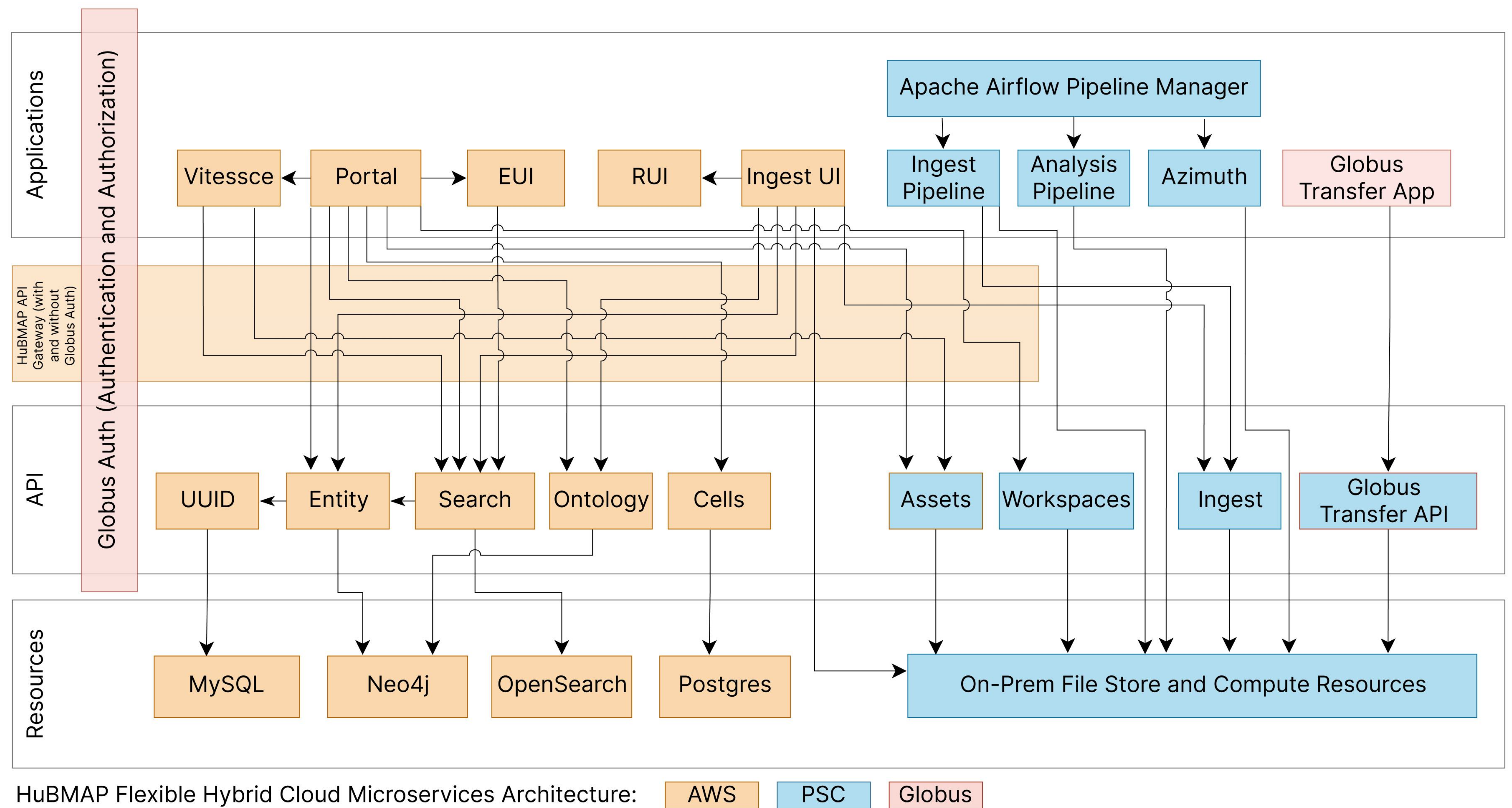

Supplemental Figure 1. Hybrid Cloud Microservices System Architecture

Supplement: Supplement 1 [file media-1.zip › 1 v3.12.2024.pdf]

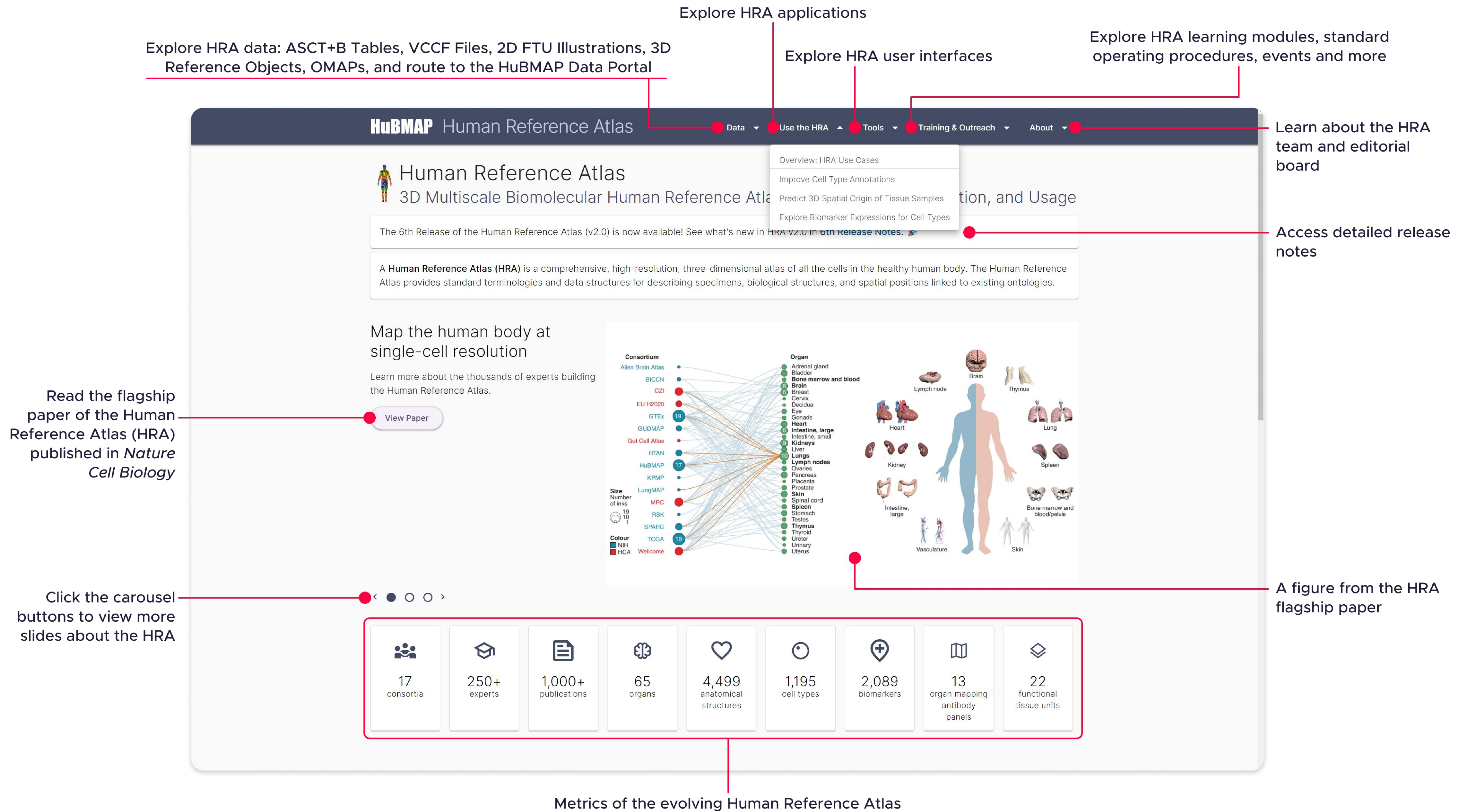

Supplemental Figure 3. Human Reference Atlas Portal

Supplement: Supplement 1 [file media-1.zip › 3 v3.12.2024.pdf]

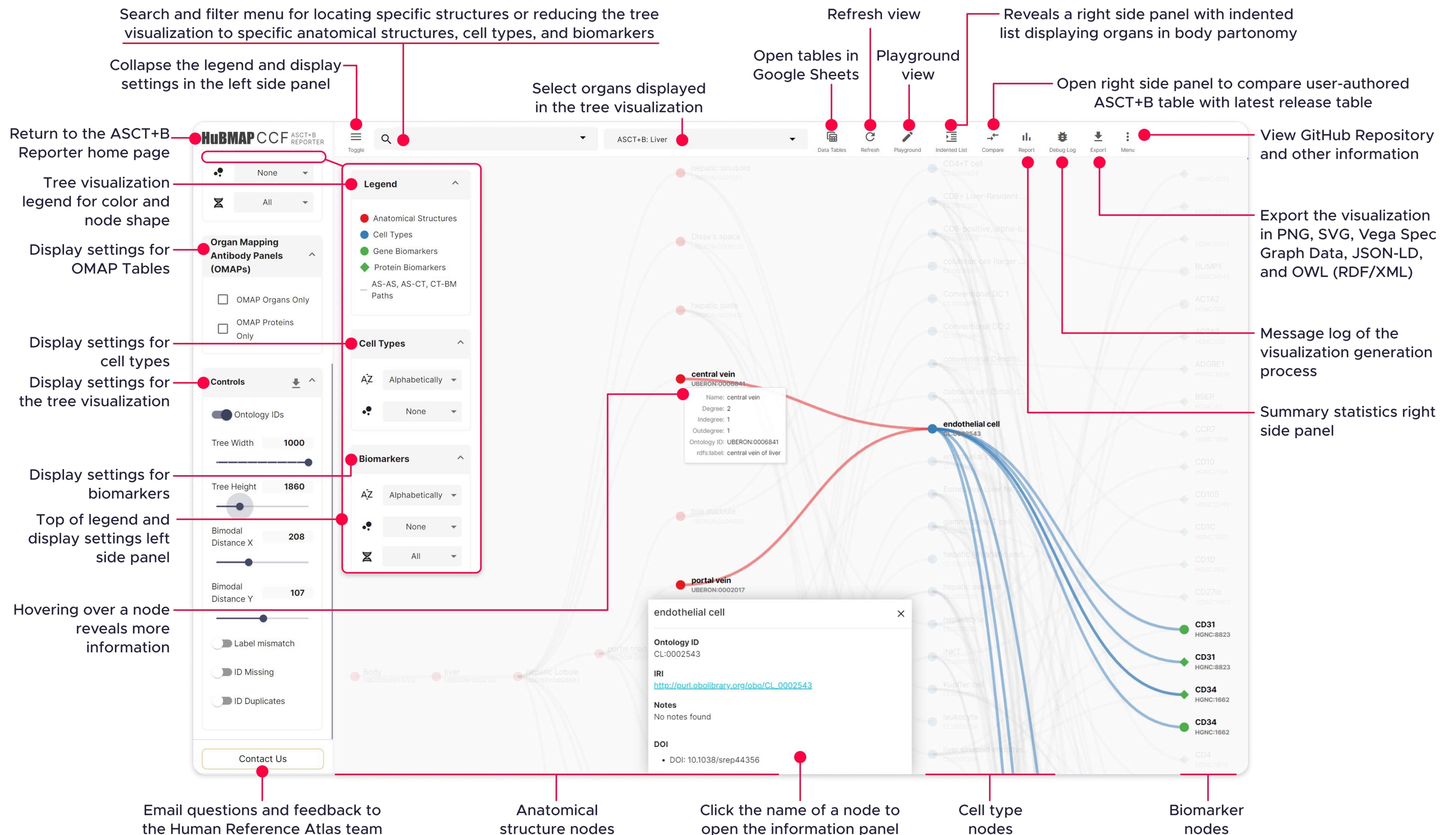

Supplemental Figure 4: ASCT+B Reporter User Interface

Supplement: Supplement 1 [file media-1.zip › 4 v3.12.2024.pdf]

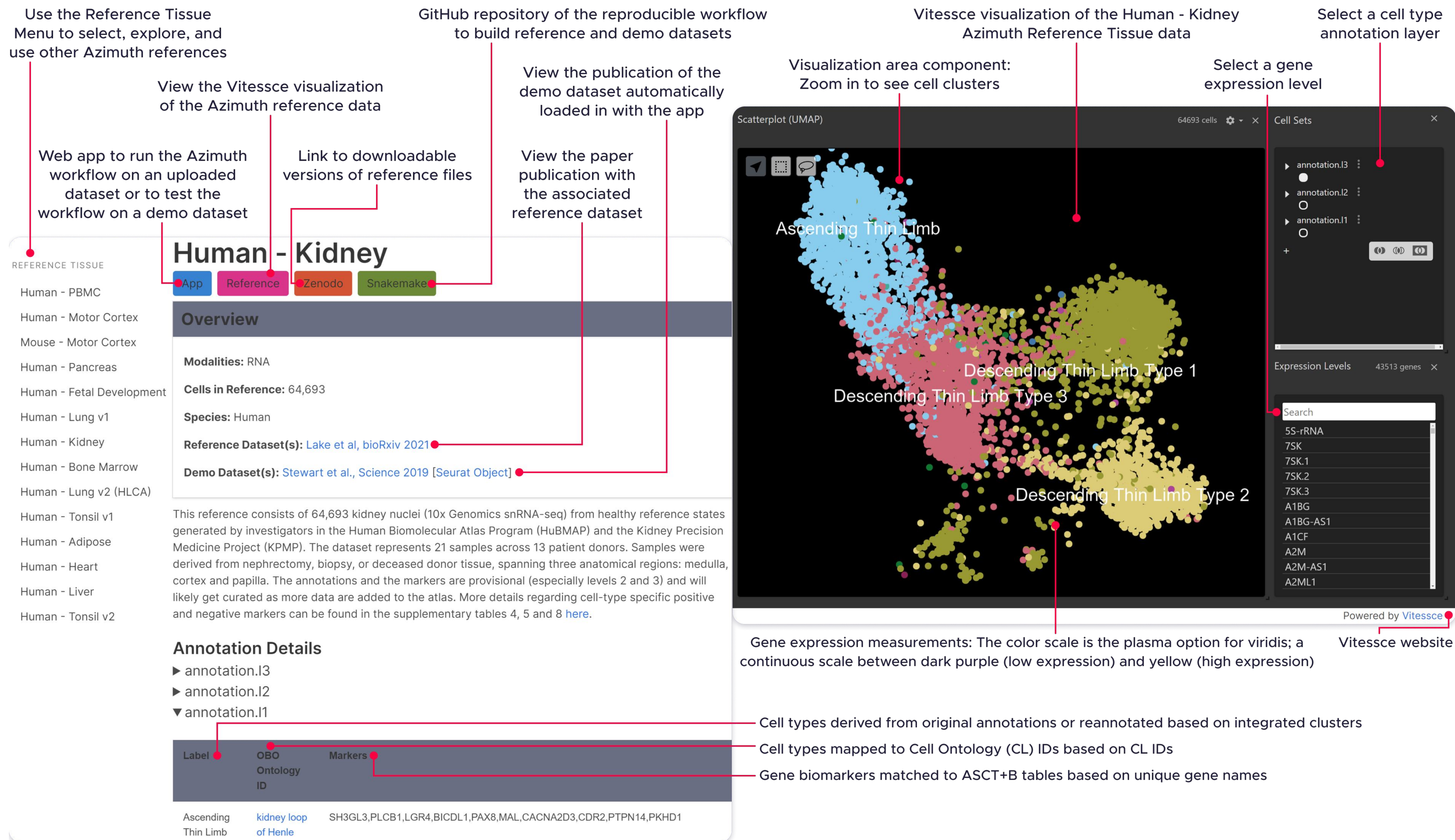

Supplemental Figure 5. Azimuth Portal and Reference Explorer User Interface

Supplement: Supplement 1 [file media-1.zip › 5 v3.12.2024.pdf]

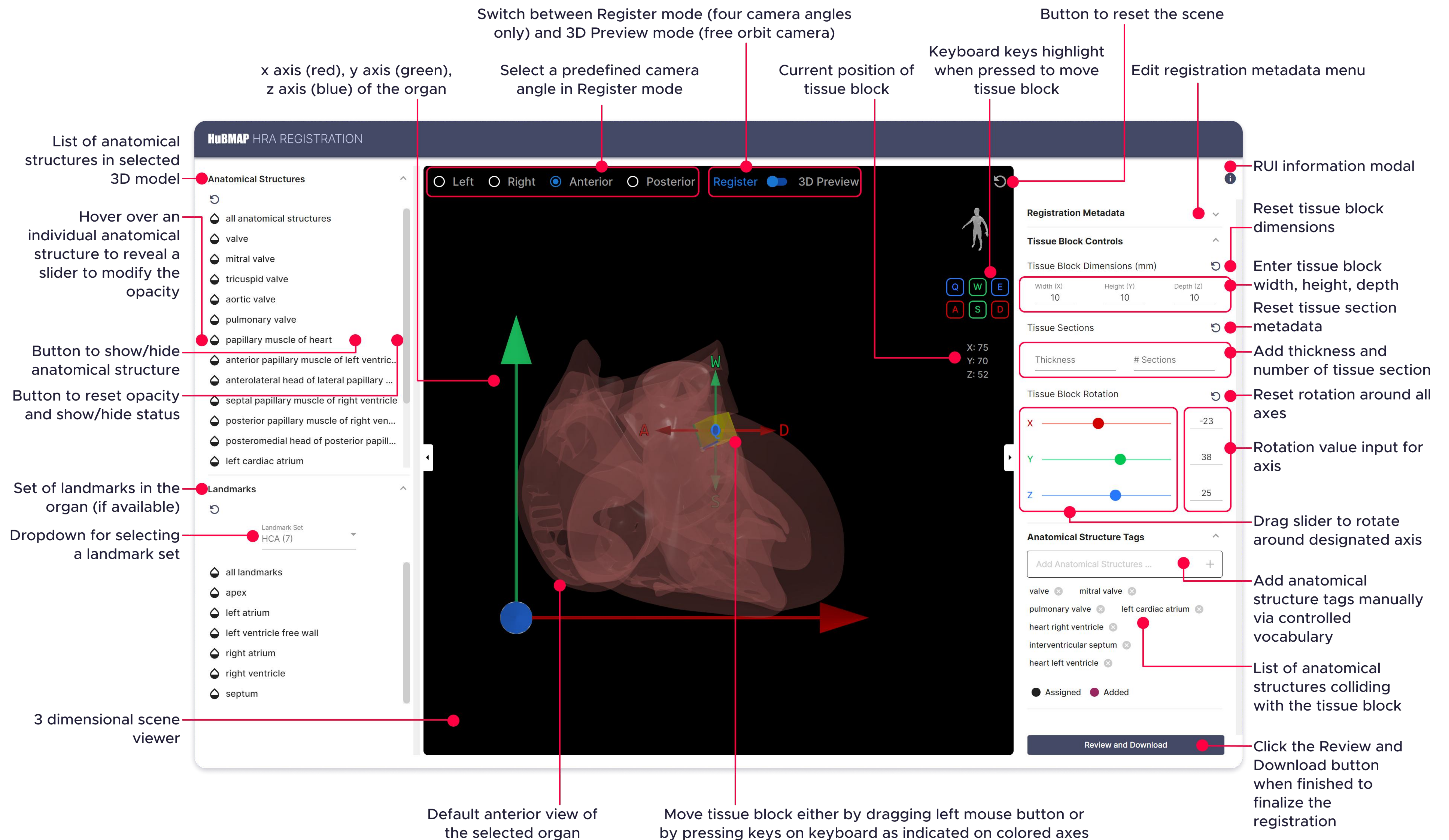

Supplemental Figure 6: Registration User Interface (RUI)

Supplement: Supplement 1 [file media-1.zip › 6 v3.12.2024.pdf]

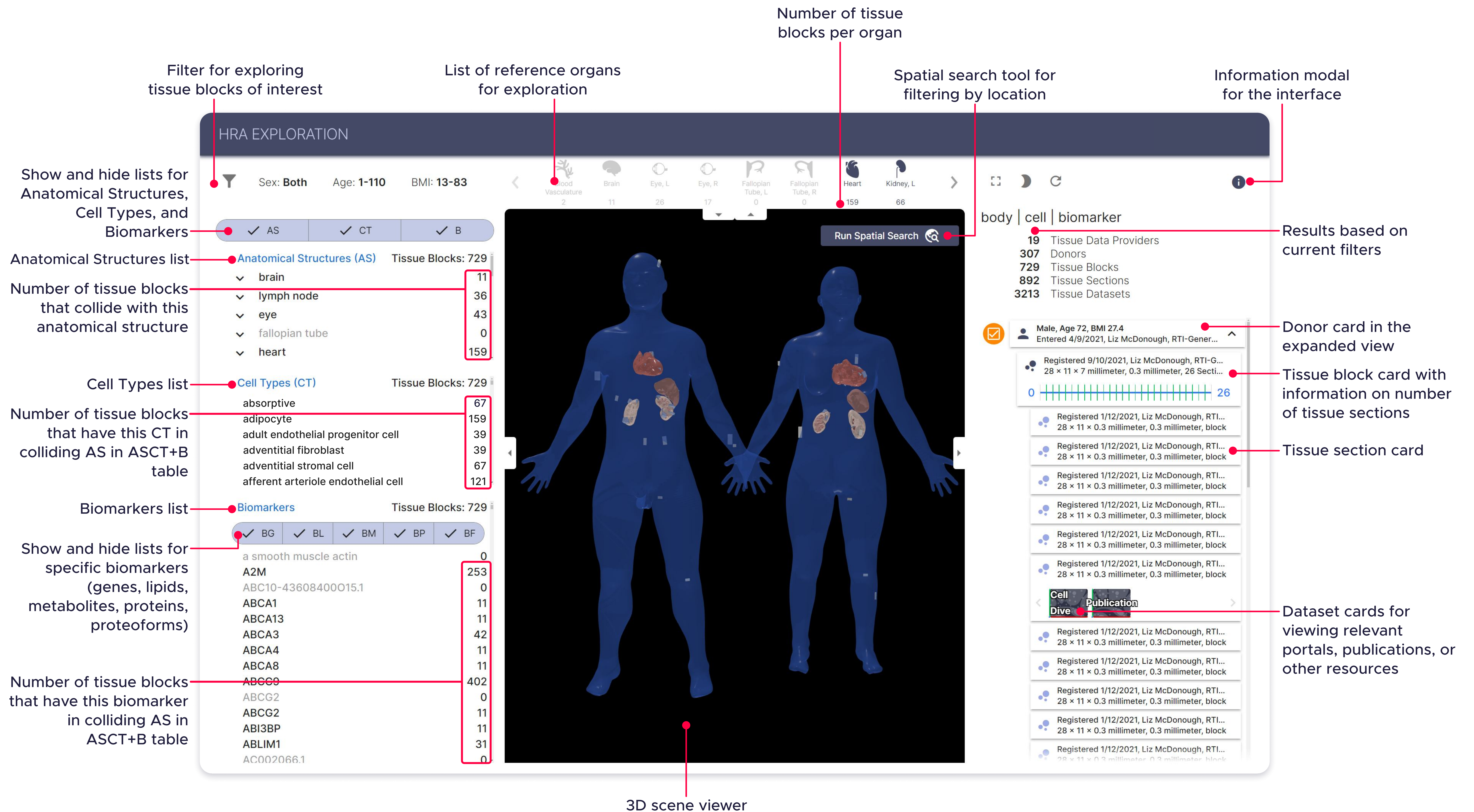

Supplemental Figure 7: Exploration User Interface (EUI)

Supplement: Supplement 1 [file media-1.zip › 7 v3.12.2024.pdf]

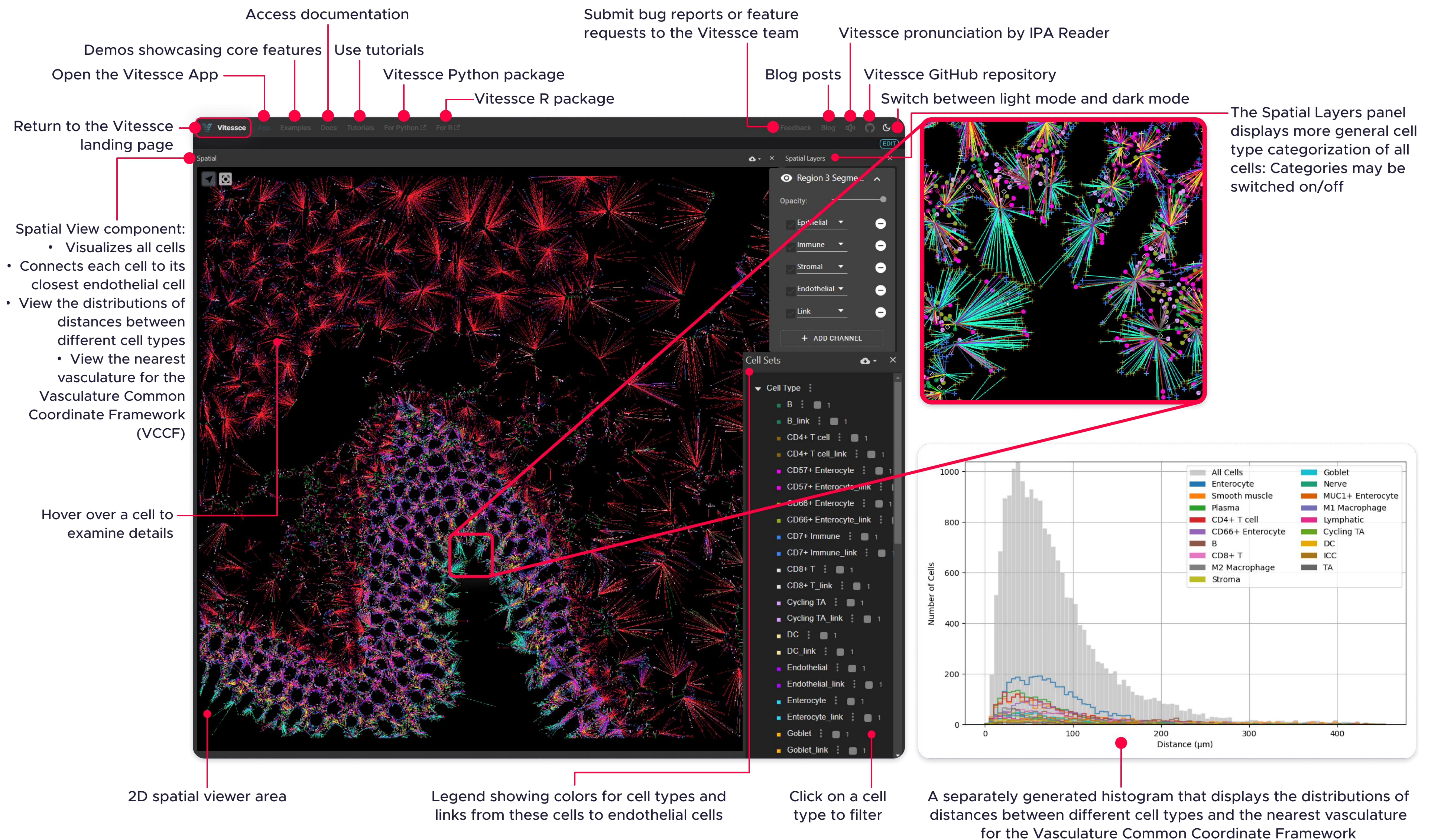

Supplement: Supplement 1 [file media-1.zip › 9 v3.12.2024.pdf]

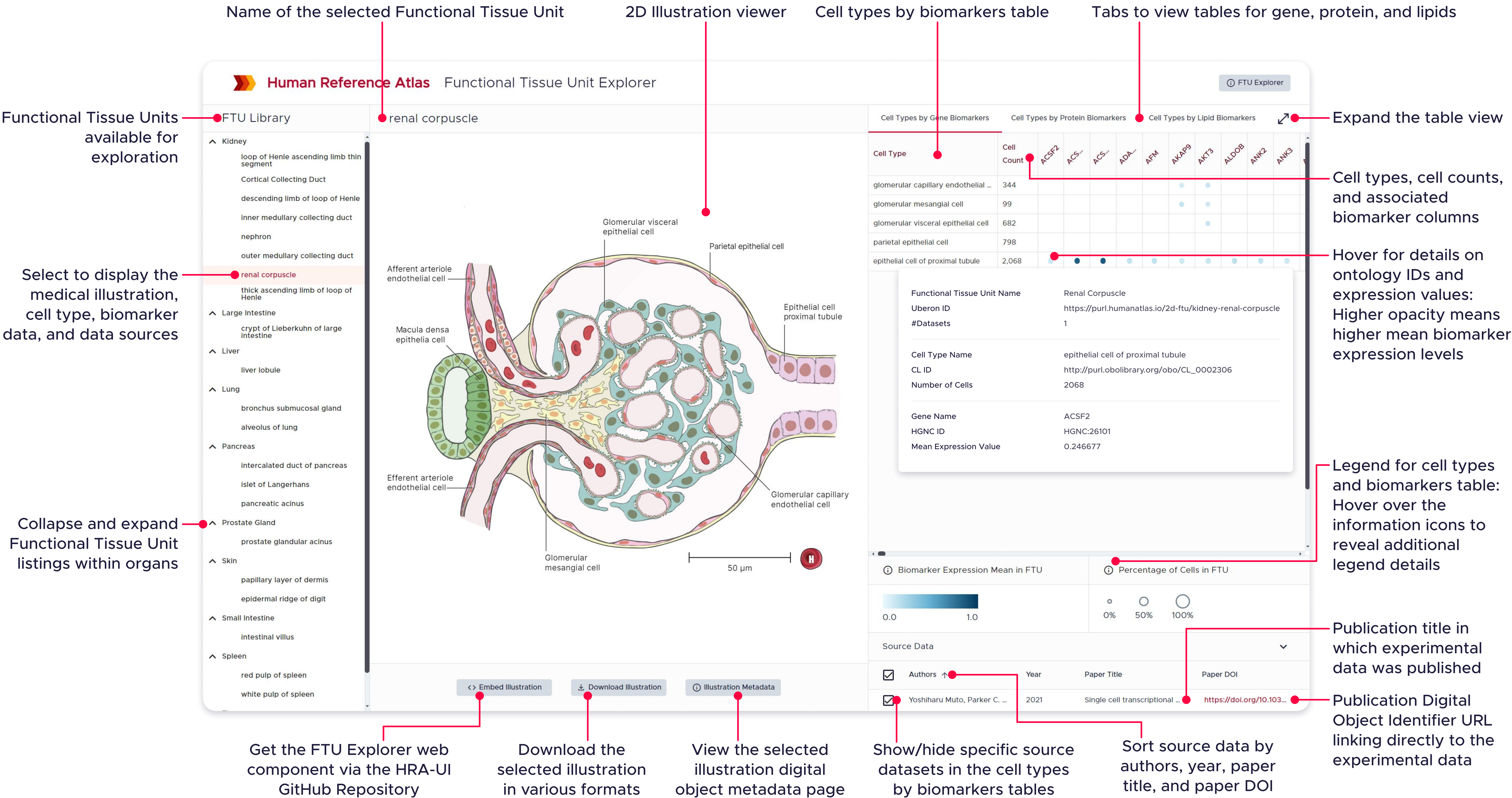

Supplemental Figure 10: Interactive FTU Explorer

Supplement: Supplement 1 [file media-1.zip › 10 v8.12.2024 v2.pdf]

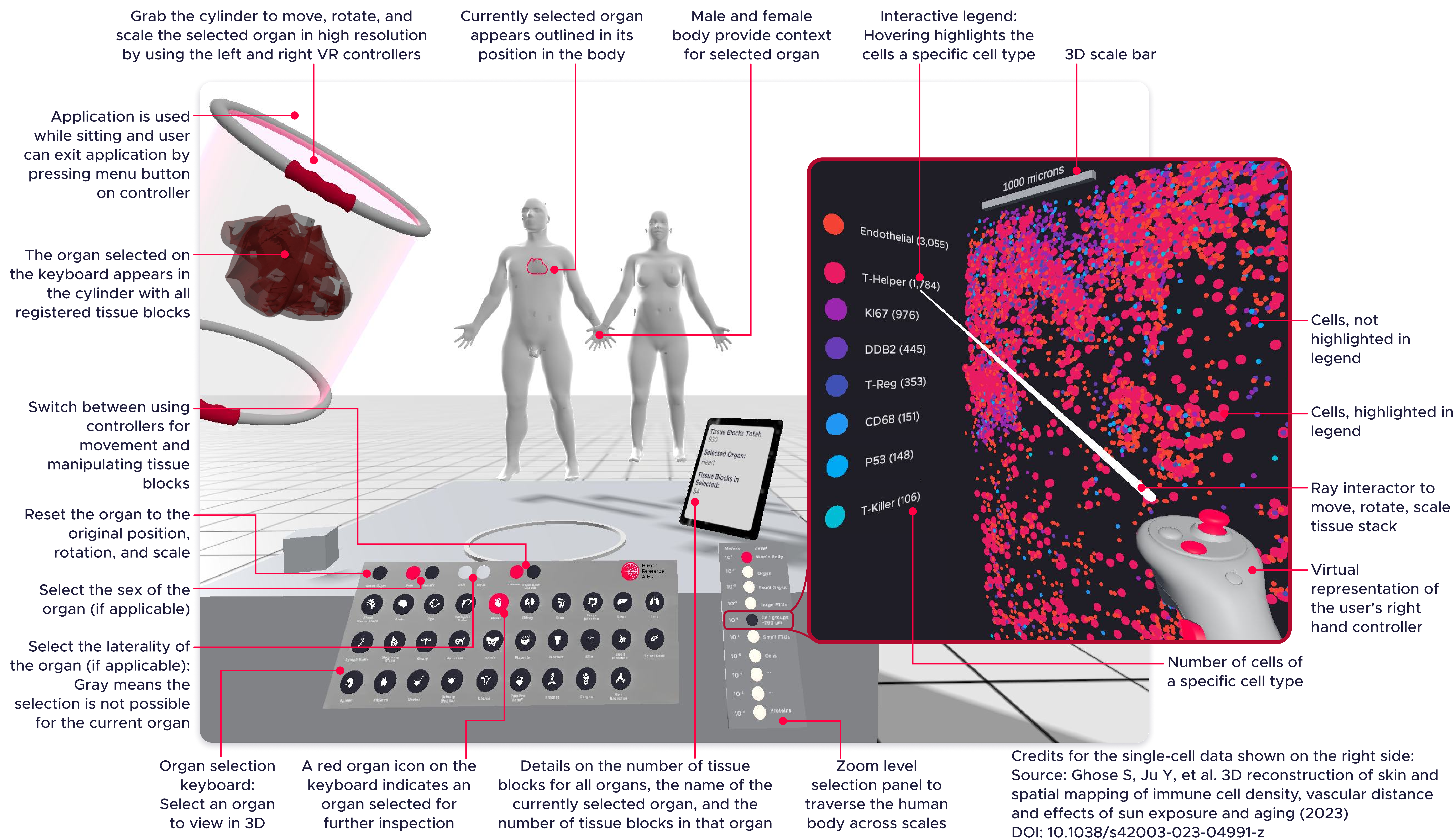

**Supplemental Figure 11: HRA Organ Gallery**

Supplement: Supplement 1 [file media-1.zip › 11 v7.26.2024.pdf]

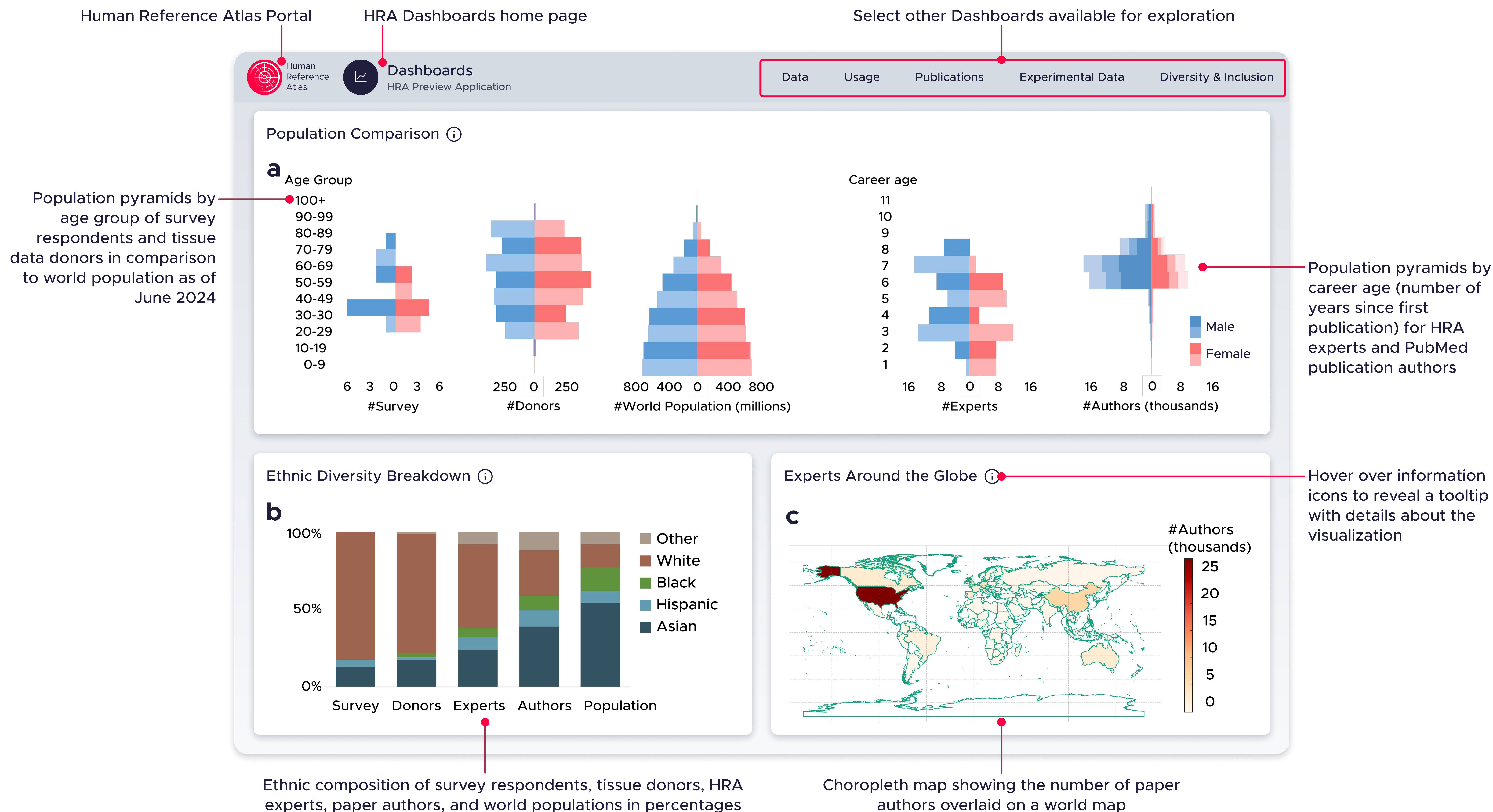

Supplemental Figure 15. HRA Equity Dashboard

Supplement: Supplement 1 [file media-1.zip › 15 v7.15.2024.pdf]

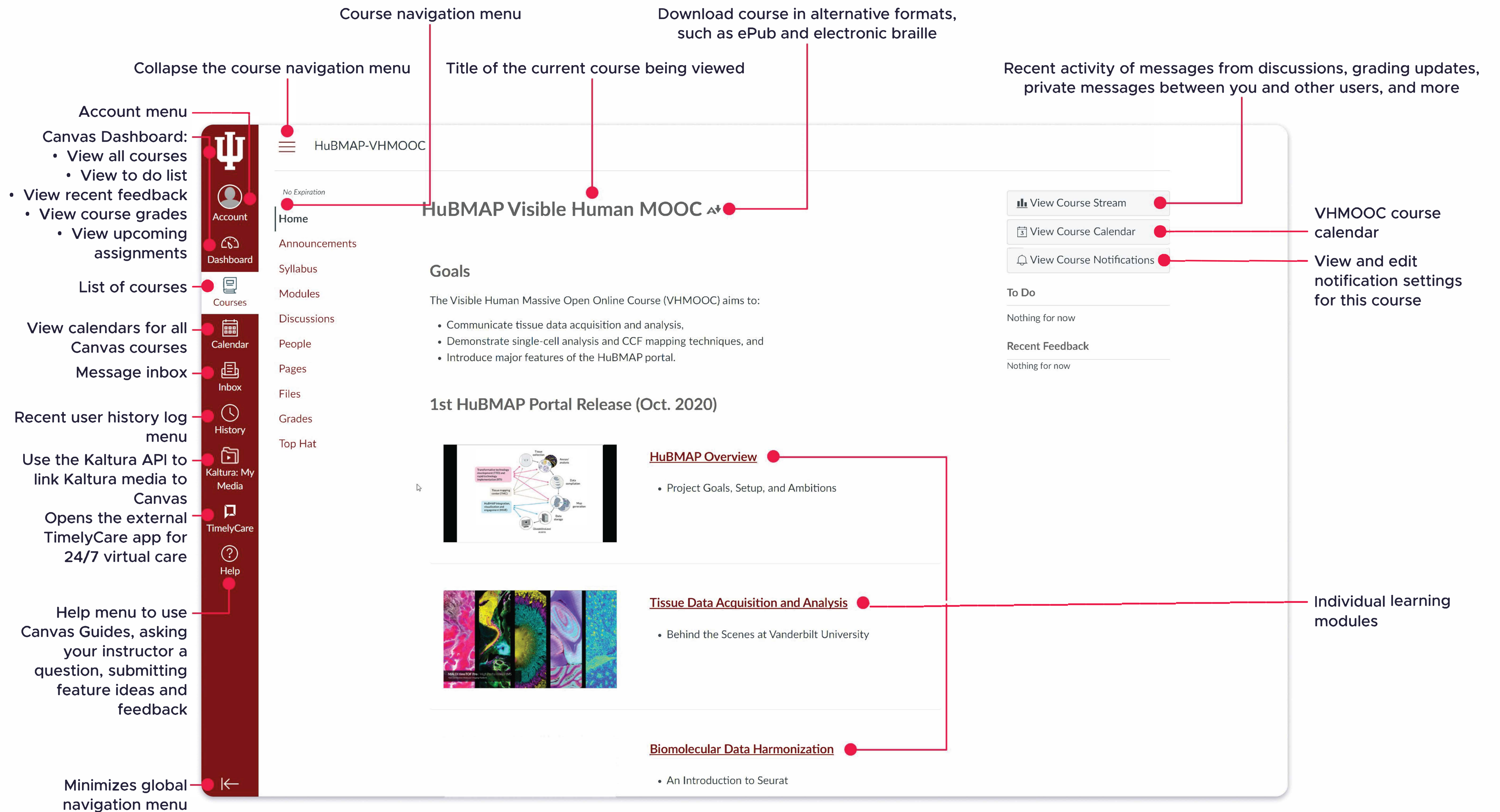

Supplemental Figure 16: Visible Human Massive Open Online Course (MOOC)

Supplement: Supplement 1 [file media-1.zip › 16 v3.12.2024.pdf]
