# Supplementary material for: Human BioMolecular Atlas Program (HuBMAP): 3D Human Reference Atlas Construction and Usage
Source: bioRxiv. 2024 Aug 14:2024.03.27.587041. Preprint. [Version 3] doi: 10.1101/2024.03.27.587041 (PMC11142047; doi:10.1101/2024.03.27.587041)
Supplement: 2 [file NIHPP2024.03.27.587041v3-supplement-2.pdf]

bioRxiv preprint doi: https://doi.org/10.1101/2024.03.27.587041; this version posted August 14, 2024. The copyright holder for this preprint (which was not certified by peer review) is the author/funder, who has granted bioRxiv a license to display the preprint in perpetuity. It is made available under aCC-BY 4.0 International license.

Supplemental Tables

Supplemental Table 1: Experimental Data, HRA Data and Cell Type Annotation by Organ. Number of sc/snRNA-seq datasets per organ vs. number of 3D reference organs plus anatomical structure and unique cell types in ASCT+B tables vs. number of cell types that different cell type annotation tools can assign.

		ASCT+B and 3D Reference Organs			Cell Type Annotation Tools		
**Organ**	**Datasets with H5AD file**	**#AS in 3D (male+female)**	**#AS**	**#CT**	**Azimuth**	**CellTypist**	**popV**
blood	2,501	none	1	29	41	26	18
blood vasculature	3	244	1,054	10	0	0	14
bone marrow	131	none	1	47	43	39	14
brain*	656	210	188	625	0	0	0
brain > motor cortex*	Part of 656	4**	1	139	20	0	0
brain > hippocampus*	Part of 656	12**	4	45	0	20	0
breast/mammary gland	1	18	3	10	0	0	14
eye	192	96	39	55	0	0	27
heart	254	34	52	28	25	49	6
kidney	207	116	61	70	58	34	0
large intestine	140	22	54	58	0	0	16
liver	70	46	17	30	25	35	12
lung	531	141	54	74	78	71	36
lymph node***	16	16	34	45	0	28	22
pancreas	14	11	31	30	11	9	14
prostate gland	34	18	13	19	0	0	13
skin of body	53	2	15	35	0	23	21
small intestine	177	23	39	34	0	0	16
spleen	39	12	37	59	0	32	22
thymus	47	6	18	50	0	0	21
trachea	29	8	20	17	0	0	18
urinary bladder	0	15	16	15	0	0	14
uterus	23	10	61	18	0	0	13
**Total (sum, not unique)**	**5,118**	**1,064**	**1,810**	**1,542**	**301**	**366**	**331**

* Azimuth and CellTypist focus on the motor cortex and hippocampus, respectively. The 3D Reference Object and ASCT+B Table of the brain in the HRA focus on the entire organ.
** Primary motor cortex is part of the precentral gyrus and hippocampus has three (head, body, tail) 3D reference objects in each hemisphere in male and female.
*** The 3D lymph node is generic and was placed in the mesenteric region as all HuBMAP data is from that region.

bioRxiv preprint doi: https://doi.org/10.1101/2024.03.27.587041; this version posted August 14, 2024. The copyright holder for this preprint (which was not certified by peer review) is the author/funder, who has granted bioRxiv a license to display the preprint in perpetuity. It is made available under aCC-BY 4.0 International license.

Supplemental Table 2: Primary and Secondary HRA Data Repositories. Color coding: Data used in HuBMAP Data Portal in blue, HRA Portal in red, in demonstration Previews in yellow, external code in white.

Object Type	Primary data repository	Data mirrors
UBKG	https://ubkg.docs.xconsortia.org	
HuBMAP Ontology API	https://smart-api.info/ui/d10ff85265d8b749fbe3ad7b51d0bf0a	
CCF.OWL v2.3.0	https://lod.humanatlas.io/graph/ccf	https://www.ebi.ac.uk/ols/ontologies/ccf<br>https://bioportal.bioontology.org/ontologies/CCF<br>https://ontobee.org/ontology/CCFO
HRA KG	https://lod.humanatlas.io	
HRA ASCT+B Tables	https://humanatlas.io/asctb-tables	https://commons.datacite.org/repositories/8orcvek<br>https://datasetsearch.research.google.com/search?query=HuBMAP
HRA OMAPs	https://humanatlas.io/omap	https://cdn.humanatlas.io/hra-releases
AVRs	https://avr.hubmapconsortium.org	https://cdn.humanatlas.io/hra-releases
HRA 2D references	https://humanatlas.io/2d-ftu-illustrations	https://cdn.humanatlas.io/hra-releases
HRA 3D references	https://humanatlas.io/3d-reference-library	https://cdn.humanatlas.io/hra-releases<br>NIH3D: https://3d.nih.gov/collections/hra<br>EMBL: https://www.ebi.ac.uk/ols4/ontologies/ccf, e.g., brain is at http://purl.obolibrary.org/obo/UBERON_0000955
HRA VCCF	https://humanatlas.io/vccf	https://cdn.humanatlas.io/hra-releases
Azimuth	https://azimuth.hubmapconsortium.org	
CellTypist	https://www.celltypist.org/models	https://pypi.org/project/celltypist/
popV	https://github.com/YosefLab/PopV	https://zenodo.org/records/7580707
FTU Segmentation training data	HuBMAP Collection doi:10.35079/hbm925.sgxl.596	https://zenodo.org/records/7729610<br>https://zenodo.org/records/7545745
HRApop	https://lod.humanatlas.io/graph/hra-pop/latest/	
HRApop Atlas Enriched Dataset Graph	https://cdn.humanatlas.io/digital-objects/graph/hra-pop/v0.10.2/assets/atlas-enriched-dataset-graph.jsonld	
HRApop AS Cell Summaries	https://cdn.humanatlas.io/digital-objects/graph/hra-pop/v0.10.2/assets/atlas-as-cell-summaries.jsonld	

bioRxiv preprint doi: https://doi.org/10.1101/2024.03.27.587041; this version posted August 14, 2024. The copyright holder for this preprint (which was not certified by peer review) is the author/funder, who has granted bioRxiv a license to display the preprint in perpetuity. It is made available under aCC-BY 4.0 International license.

"Hierarchical cell type populations within FTUs" data	https://portal.hubmapconsortium.org/browse/publication/77ab35880329b5932380104aa58795a4	https://doi.org/10.5061/dryad.pk0p2ngrf<br>https://doi.org/10.5061/dryad.76hdr7t1p (Part 1)<br>https://doi.org/10.5061/dryad.gmsbcc2sq (Part 2)
"Perivascular immune cells in lung" data	https://drive.google.com/drive/folders/1SPbN6_0C-mVWbfE5r2OTVZLMxBjiEKnI?usp=sharing	

Supplemental Table 3: HRA Code Repositories. Color coding: Data used in HuBMAP Data Portal in blue, HRA Portal in red, in demonstration Previews in yellow, external code in white.

Code Type and Name	GitHub Repository
**Data processing**	
QuPath Manual Segmentation Tool (0.5.1)	https://qupath.github.io
Azimuth (0.4.6)	https://github.com/hubmapconsortium/azimuth-annotate
CellTypist (1.6)	https://github.com/Teichlab/celltypist
popV (0.9)	https://github.com/YosefLab/PopV
van Valen Tools	https://github.com/vanvalenlab?q=deep
van Valen's Cell Type Annotation (HuBMAP internal)	https://github.com/hubmapconsortium/deepcelltypes-hubmap
HuBMAP sc/snRNA-seq and CODEX pipelines	https://github.com/hubmapconsortium/salmon-rnaseq<br>https://github.com/hubmapconsortium/codex-pipeline
3DCellComposer (1.2)	https://github.com/murphygroup/3DCellComposer
CytoSpatio (1.0.0)	https://github.com/murphygroup/CytoSpatio
PanelOptimizer	https://github.com/murphygroup/CODEXPanelOptimization
CellSegmentationEvaluator (1.5)	https://github.com/murphygroup/CellSegmentationEvaluator
FTU segmentation via Kaggle #1-2 Competitions	https://github.com/hubmapconsortium/pas-ftu-segmentation-pipeline<br>https://github.com/cns-iu/hra-multiftu-segmentation-pipeline
Cell Neighborhood Analysis	https://github.com/HickeyLab/Hierarchical-Tissue-Unit-Annotation
STELLAR	https://github.com/snap-stanford/stellar
STalign (1.0.1)	https://github.com/JEFworks-Lab/STalign
HRA Dataset Graphs Library (1.0)	https://github.com/hubmapconsortium/hra-rui-locations-processor
HRA Digital Object Processor	https://github.com/hubmapconsortium/hra-do-processor
HRA Workflows	https://github.com/hubmapconsortium/hra-workflows
HRA Workflows Runner	https://github.com/hubmapconsortium/hra-workflows-runner
HRA Multi-LOD Renderer (1.0.0)	https://github.com/cns-iu/hra-multi-lod

bioRxiv preprint doi: https://doi.org/10.1101/2024.03.27.587041; this version posted August 14, 2024. The copyright holder for this preprint (which was not certified by peer review) is the author/funder, who has granted bioRxiv a license to display the preprint in perpetuity. It is made available under aCC-BY 4.0 International license.

HRA AMAP	https://github.com/cns-iu/hra-amap
VCCF Visualizations	https://github.com/cns-iu/hra-vccf-cell-distance-visualizations
Vascular distance for 3D reconstruction of skin and spatial mapping of immune cell density	https://github.com/hubmapconsortium/vccf-visualization-2022
EBI ASCT+B Table Validation (2024-07-11)	https://github.com/hubmapconsortium/ccf-validation-tools
HRA 3D Reference Organ Validation	https://github.com/hubmapconsortium/hra-ref-organ-validation
**API for data access**	
HuBMAP Cells API	https://github.com/hubmapconsortium/cross_modality_query
HuBMAP Cells API (Python Client) (0.0.11)	https://github.com/hubmapconsortium/hubmap-api-py-client
HuBMAP Entity API (2.3.15)	https://github.com/hubmapconsortium/entity-api
HuBMAP Ingest API (2.3.17)	https://github.com/hubmapconsortium/ingest-api
HuBMAP Search API (3.3.12)	https://github.com/hubmapconsortium/search-api
API and SPARQL queries using the HRA-KG	https://github.com/hubmapconsortium/ccf-grlc
Ontology API for HuBMAP and SenNet applications (2.0.3) (extends UBKG API [2.1.4])	https://github.com/x-atlas-consortia/hs-ontology-api<br>https://github.com/x-atlas-consortia/ubkg-api
HuBMAP UUID API (2.4.3)	https://github.com/x-atlas-consortia/uuid-api
HRA API (0.8.0)	https://github.com/x-atlas-consortia/hra-api
Tissue Block Annotation (1.0.0)	https://github.com/hubmapconsortium/hra-tissue-block-annotation
**User interfaces**	
HuBMAP Consortium	https://hubmapconsortium.org
HuBMAP Data Portal (0.102.5)	https://github.com/hubmapconsortium/portal-ui,<br>https://portal.hubmapconsortium.org
HRA Portal	https://github.com/hubmapconsortium/hra-ui/tree/main/apps/humanatlas.io
Vitessce (3.4.6)	https://github.com/vitessce/vitessce
ASCT+B Reporter (2.8)	https://github.com/hubmapconsortium/hra-ui
EUI (3.8)	https://github.com/hubmapconsortium/hra-ui
RUI (3.8)	https://github.com/hubmapconsortium/hra-ui
FTU Explorer (0.5.0)	https://github.com/hubmapconsortium/hra-ui
HRA Organ Gallery (0.11.2)	https://github.com/cns-iu/hra-organ-gallery-in-vr
HRA Data Dashboard (0.1.0)	https://github.com/hubmapconsortium/hra-data-dashboard
VCCF Cell Distance Visualizations (0.1.0)	https://github.com/hubmapconsortium/hra-ui
Learn about the HRA through scrollytelling!	https://github.com/cns-iu/hra-scrollytelling
HRA Pilot Previews	https://github.com/hubmapconsortium/hra-previews
**Data / Ontologies**	
HuBMAP Ontology (CCF v1.x)	https://github.com/hubmapconsortium/hubmap-ontology

bioRxiv preprint doi: https://doi.org/10.1101/2024.03.27.587041; this version posted August 14, 2024. The copyright holder for this preprint (which was not certified by peer review) is the author/funder, who has granted bioRxiv a license to display the preprint in perpetuity. It is made available under aCC-BY 4.0 International license.

Human Reference Atlas Knowledge Graph (HRA-KG)	https://github.com/hubmapconsortium/hra-kg
HRA Vasculature CCF	https://github.com/hubmapconsortium/hra-vccf
HRA Vocabulary (2.5.10)	https://github.com/hubmapconsortium/hra-vocab
HRApop (0.10.2)	https://github.com/x-atlas-consortia/hra-pop
HRAlit (0.5)	https://github.com/cns-iu/hra-literature
