# Supplementary material for: Human BioMolecular Atlas Program (HuBMAP): 3D Human Reference Atlas Construction and Usage
Source: bioRxiv. 2024 May 22:2024.03.27.587041. Preprint. [Version 2] doi: 10.1101/2024.03.27.587041 (PMC11142047; doi:10.1101/2024.03.27.587041)
Supplement: Supplementary file 2 [file media-1.zip › SFigs 5.21.2024/15 v3.12.2024.pdf]

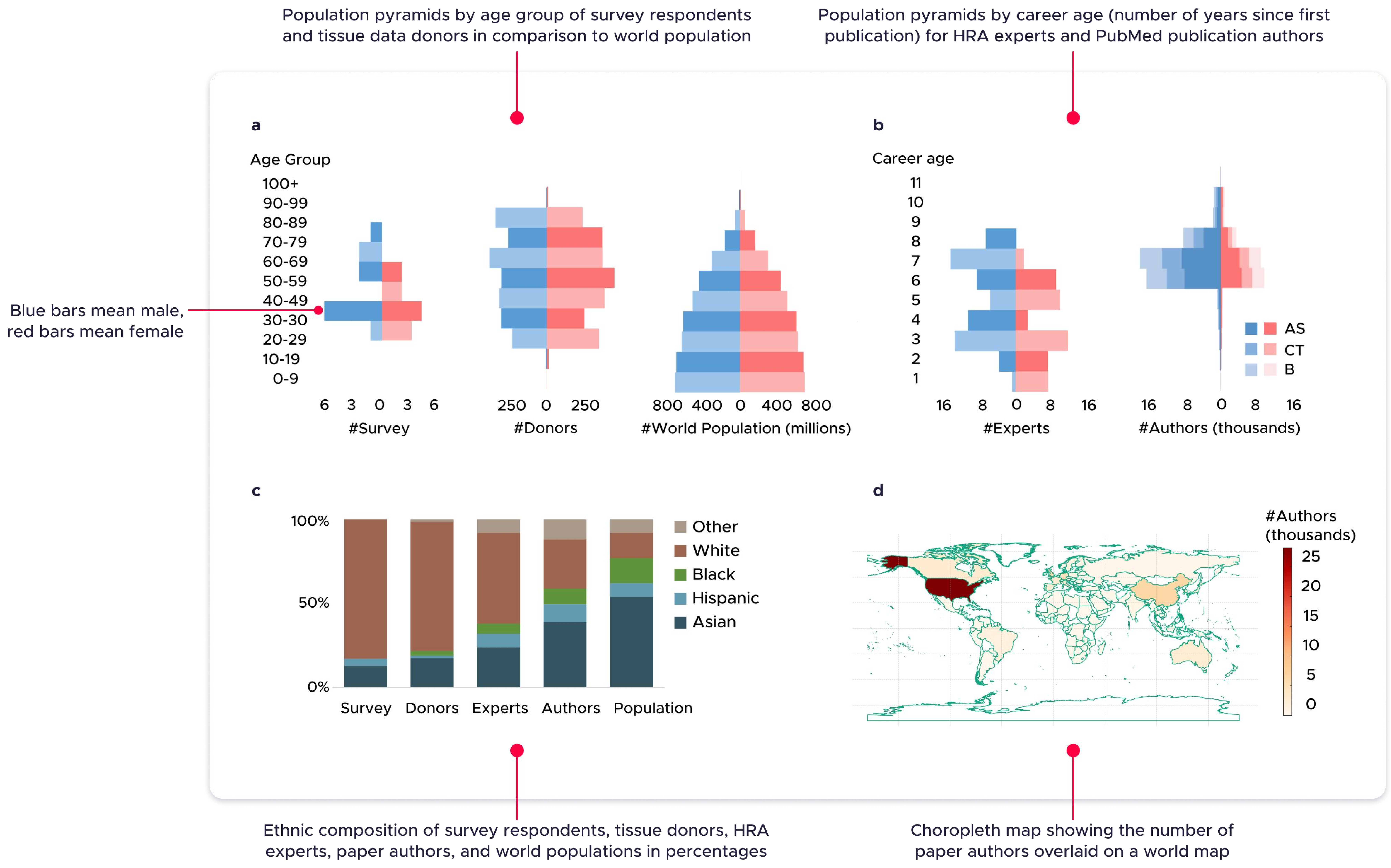

Supplemental Figure 15. HRA Equity Dashboard
